# β-Cyclodextrin-Complexed 1,8-Cineole Attenuates Motor Deficits and Preserves Tyrosine Hydroxylase Immunoreactivity in a Reserpine-Induced Mouse Model of Parkinsonism

**DOI:** 10.1007/s12035-026-06262-4

**Published:** 2026-10-15

**Authors:** Luan Araújo-Monteiro, Beatriz Soares-Silva, Patrícia Santos Carvalhinho-Lopes, Valéria Cunha Moreira, Barbara Vitória de Campos Cruz Binatto, Ellen Games Jacob Pereira, Ana Paula Nascimento de Lima, Lucindo José Quintans-Junior, José Ronaldo dos Santos, Alessandra Mussi Ribeiro

**Affiliations:** 1 Department of Biosciences – UNIFESP, Federal University of São Paulo, Rua Silva Jardim, 136, Edifício Central, Santos, São Paulo CEP 11.015-020 Brazil; 2 Department of Physiology, Federal University of Sergipe, São Cristovão, Brazil; 3 Department of Biosciences, Federal University of Sergipe, Itabaiana, Brazil

**Keywords:** 1,8-Cineole, Parkinson’s disease, Reserpine, Tyrosine hydroxylase, α-Synuclein

## Abstract

Parkinson’s disease (PD) is a progressive neurodegenerative disorder characterized by dopaminergic dysfunction and motor impairment. Natural compounds have emerged as promising candidates for neuroprotection and symptomatic relief in experimental models of PD. This study investigated the effects of β-cyclodextrin-complexed 1,8-cineole (CIN) in a reserpine (RES)-induced mouse model of parkinsonism. Male mice were treated with RES (0.1 mg/kg, s.c., every 48 h) and/or CIN (5 mg/kg, p.o., once daily) for 30 days. Behavioral assessments evaluated motor, sensorimotor, and locomotor function. Tyrosine hydroxylase (TH) and α-synuclein immunoreactivity were assessed by immunohistochemistry. Repeated RES administration induced progressive motor and sensorimotor impairments and reduced TH immunoreactivity in mesencephalic dopaminergic nuclei. CIN treatment significantly reduced catalepsy, vacuous chewing movements, and chin tremors and improved sensorimotor performance in the adhesive removal test. These behavioral effects were accompanied by preservation of TH immunoreactivity in the substantia nigra pars compacta (SNpc) and ventral tegmental area (VTA). However, CIN did not significantly alter hippocampal α-synuclein immunoreactivity. These findings indicate that β-cyclodextrin-complexed CIN attenuates motor and sensorimotor deficits and is associated with preservation of dopaminergic markers in a RES-induced mouse model of parkinsonism. These results support further investigation of CIN as a neuroactive compound in experimental models of PD.

## Introduction

Neurodegenerative diseases (NDs) comprise a group of disorders characterized by progressive neuronal loss, leading to cognitive and motor impairments and other comorbidities that profoundly reduce quality of life [1]. Parkinson’s disease (PD) is the second most prevalent ND and is neuropathologically characterized by dopaminergic neurodegeneration and the presence of Lewy bodies [2]. Its etiology is multifactorial, and the global increase in PD prevalence is closely associated with rising life expectancy and the growing burden of age-related disorders [3, 4]. PD is clinically characterized by motor symptoms such as resting tremor, bradykinesia, and postural instability, which typically emerge only after approximately 70% of dopaminergic neurons have been lost [5]. However, pathological changes begin earlier, during the prodromal phase, when dysregulated reactive oxygen species (ROS) production, oxidative stress, and chronic neuroinflammation may contribute to disease progression [6, 7]. Within the basal ganglia, degeneration of dopaminergic neurons in the substantia nigra pars compacta (SNpc) leads to dopamine depletion in the striatum and contributes to motor deficits observed in PD [8, 9]. Another major neuropathological hallmark of PD is the presence of Lewy bodies, intracellular inclusions enriched in α-synuclein (α-Syn). An important study by Spillantini et al. [10] identified α-Syn as a major component of Lewy bodies, establishing its central role in PD pathology. Although its causal contribution remains incompletely understood, α-Syn aggregates have been associated with neuroinflammation, mitochondrial dysfunction, impaired synaptic transmission, and neuronal vulnerability [11–13]. In addition to the nigrostriatal system, α-Syn-related pathology has been reported in extranigral regions, including cerebellum, striatum, neocortex, and hippocampus. These alterations may contribute to non-motor symptoms of PD, including cognitive and neuropsychiatric symptoms [14].

Beyond motor deficits, patients with PD frequently present with early non-motor symptoms, including cognitive decline, neuropsychiatric disturbances, sleep alterations, and autonomic dysfunction. These features emerge as the disease affects extranigral dopaminergic, cholinergic, and serotonergic pathways [14, 15]. Chaudhuri and Schapira [16] provided one of the most comprehensive descriptions of these manifestations, highlighting their clinical significance. Dysfunction of the locus coeruleus (LC), a major nucleus enriched in cannabinoid, serotonergic, and cholinergic receptors, has been implicated in PD progression. Although LC neurons are not dopaminergic, their degeneration and widespread connectivity further support the view that PD extends beyond dopaminergic circuits [17–19].

Despite intensive research, PD remains incurable, and available therapies primarily provide symptomatic relief [20]. Current pharmacological strategies include monoamine oxidase-B (MAO-B) inhibitors, catechol-O-methyltransferase (COMT) inhibitors, and dopamine agonists [21]. Levodopa (L-DOPA), originally identified in *Mucuna pruriens* plant, remains the gold-standard symptomatic therapy and acts as a dopamine precursor. Although highly effective in the early stages, long-term administration is frequently associated with motor complications, treatment resistance, and reduced efficacy, ultimately limiting its sustained clinical benefits [22–24].

Natural products have therefore attracted attention as potential sources of novel therapeutics. Many natural compounds are relatively accessible and may exhibit biological activities relevant to neurodegenerative disorders [24]. Their antioxidant and anti-inflammatory properties are of particular interest in NDs [24–26]. Shahpiri et al. [27] reviewed their potential applications of natural compounds in PD, highlighting their promise as neuroprotective candidates. Supporting this rationale, previous studies from our group showed that *Lippia grata* essential oil improved motor and cognitive deficits in a PD model [12], and that β-cyclodextrin-complexed myrtenol exerted neuroprotective effects associated with modulation of oxidative stress-related parameters [28].

Eucalyptol (1,8-cineole), a saturated monoterpene abundant in *Eucalyptus* species and also present in *Rosmarinus officinalis* and *Cannabis sativa*, has emerged as a compound of interest [29]. Cineole exhibits anti-inflammatory [30], antioxidant [31–34], anticarcinogenic [35], and neuroprotective activities [36–38], supporting its therapeutic potential in models of NDs. Advances in biotechnology have further expanded the applications of natural products, particularly through cyclodextrin (CD) complexation. CDs are cyclic oligosaccharides that can improve the solubility, stability, and bioavailability of hydrophobic compounds. β-CDs, in particular, have been widely used to complex terpenes, providing a strategy for optimizing their pharmacological properties [39–41].

Based on this evidence, the present study investigated the effects of β-cyclodextrin-complexed 1,8-cineole in a reserpine-induced mouse model of parkinsonism by assessing motor, sensorimotor behavior and TH and α-Syn immunoreactivity in brain areas.

## Materials and Methods

### Animals

Six-month-old male *Swiss* mice (*n *= 33) were obtained from the Center for the Development of Experimental Models for Medicine and Biology (CEDEME) at the Federal University of São Paulo. Animals were housed in groups of four per cage (30 cm × 37 cm × 16 cm), under standard conditions, including controlled ventilation and temperature (24 ± 1 °C), with a 12-/12-h light-dark cycle (lights on at 7:00 a.m.), and free access to food and water. The experimental procedures were approved by the institutional Ethics Committee on Animal Use (Protocol CEUA/UNIFESP N^o^. 4876300523) and conducted in accordance with Brazilian legislation governing the use of animals in research (Law N^o^. 11,794). Every effort was made to minimize animal pain, discomfort, and suffering.

### Drugs

Reserpine (RES, #R0875), 1,8-cineole (#C80601), and β-cyclodextrin (β-CD) (#C4767) were purchased from Sigma-Aldrich. (USA). RES (0.005 g) was dissolved in 50 µL of glacial acetic acid and diluted with distilled water to a final concentration of 0.01 mg/mL in 1% glacial acetic acid. The vehicle consisted of 1% glacial acetic acid in distilled water. CIN was prepared in distilled water at 0.5 mg/mL, with distilled water serving as the vehicle. RES and its vehicle were administered subcutaneously (s.c.), whereas CIN and its vehicle were administered by oral gavage (p.o.). The essential oil of the monoterpene 1,8-cineole was complexed with β-cyclodextrin (CIN) according to the method described by Siqueira-Lima et al. [42].

### Experimental Design and General Procedures

Before the start of the experiments, animals were handled for 5 min daily for 10 consecutive days to reduce handling-related stress. Open-field (OF) activity was recorded using a camera positioned approximately 100 cm above the apparatus and, the behavioral parameters were analyzed using Any-maze® animal-tracking software (Stoelting, USA). Catalepsy, oral movements, and tactile sensitivity assessments were manually scored by an investigator blinded to the treatment allocation. All behavioral apparatus was cleaned with 70% ethanol before each session and with 5% ethanol between sessions.

**Fig. 1:**
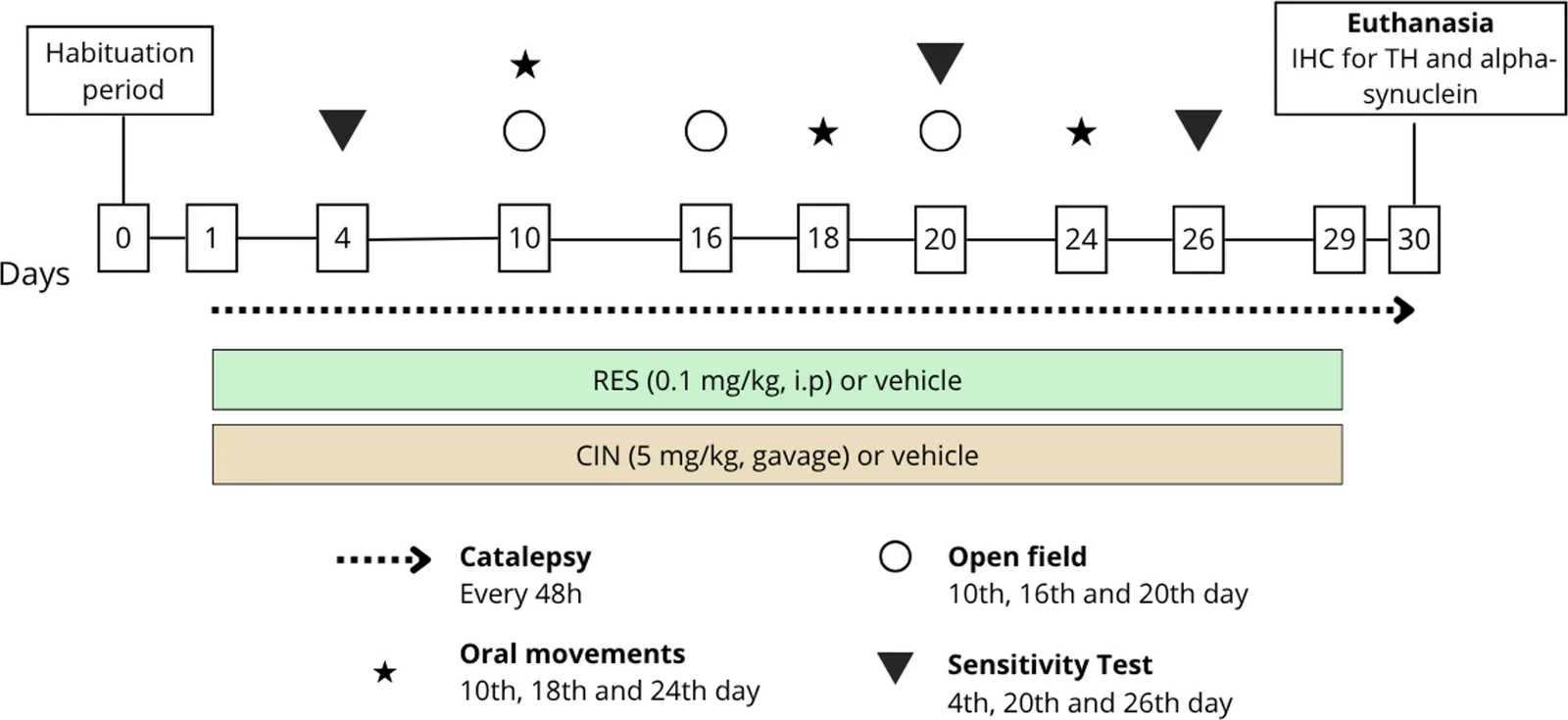
Experimental design. Schematic representation of the experimental timeline, including reserpine (RES) or vehicle administration, β-cyclodextrin-complexed 1,8-cineole (RES) or vehicle treatment, behavioral assessments, and tissue collection for immunohistochemical analyses

Mice were randomly assigned to four groups: CTL-CTL (RES vehicle + CIN vehicle, *n *= 8), CTL-CIN (RES vehicle + CIN, *n *= 9), RES-CTL (RES + CIN vehicle, *n *= 8), and RES-CIN (RES + CIN, *n *= 8). Animals received s.c. injections of RES (0.1 mg/kg) or vehicle every 48 h for 30 days (15 injections) and daily gavage of CIN (5 mg/kg) or vehicle once daily by oral gavage. Behavioral assessments, including tactile sensitivity, OF activity, catalepsy, and oral movements, were conducted during the treatment period after RES or vehicle administration. Forty-eight hours after the final RES injection, animals were anesthetized and euthanized, and their brains were collected for immunohistochemical analysis of tyrosine hydroxylase (TH) and α-synuclein (α-Syn). The experimental design is shown in Fig. 1.

### Behavioral Tests

#### Tactile Sensitivity Test

Sensorimotor deficits were assessed using the adhesive removal test [17]. Each mouse was first habituated to a transparent acrylic cylinder (20 cm in diameter × 30 cm in height) for 5 min to reduce stress and exploratory behavior within the apparatus. A 0.5 × 0.5 cm adhesive dot was then placed on the plantar surface of one forepaw. The latencies to detect and remove the adhesive were recorded, with a maximum cutoff time of 180 s. The forepaw receiving the adhesive was alternated across sessions.

#### Open Field Test

Locomotor and anxiety-like behaviors were evaluated for 5 min in a circular arena (50 cm in diameter × 40 cm in height) [43–45]. The floor of the apparatus was painted black to facilitate mouse detection by the tracking software. Each animal was placed in the center of the OF, and the analyzed parameters included total distance traveled, number of entries into the center zone, distance traveled within the center zone, and mean velocity.

#### Catalepsy Test

Motor symptoms characteristic of PD, such as postural instability and muscular rigidity, were evaluated using the catalepsy test [46]. Mice were placed with their forepaws resting on a horizontal bar elevated 5 cm above the surface. The time each animal remained in this position was recorded in seconds (up to a maximum of 180 s). Each animal underwent three trials per session, and the mean value was used for the analysis. Testing was conducted every 48 h after the first RES administration.

#### Oral Movement Assessment

Abnormal oral movements, considered behavioral correlates of orofacial dyskinesia-like activity in rodents, were quantified as described by Ricardo et al. [47]. Mice were individually placed in wire-mesh cages (20 × 15 × 25 cm) equipped with mirrors beneath and behind the cage to allow observation from all angles. The assessment comprised two consecutive 5-min phases. During the first phase, animals were habituated to the cage; during the second phase, vacuous chewing movements defined as chewing motions not directed toward any object, and chin tremors were counted.

### Perfusion and Immunohistochemistry

Twenty-four hours after the final CIN administration, mice were deeply anesthetized with ketamine (100 mg/kg), xylazine (10 mg/kg), fentanyl (0.5 mg/kg), and acepromazine (0.1 mg/kg, i.p.). Animals were transcardially perfused with approximately 200 mL of PBS (pH 7.4), followed by 200 mL of 4% paraformaldehyde (PFA) prepared in 0.1 M PBS. After perfusion, the brains were removed by craniotomy, post-fixed in the same PFA solution for 24 h, and subsequently cryoprotected in 30% sucrose solution prepared in 0.1 M PBS at 4 °C until fully cryoprotected before sectioning.

Coronal brain sections (30 μm) were obtained using a Leica CM1850 cryostat (Leica, Germany) operated at −20 °C. Sections were collected in five parallel series and stored in antifreeze solution until immunohistochemical processing. Immunohistochemistry was performed using a free-floating protocol for TH and α-Syn labeling.

Briefly, the sections were washed four times with PBS (5 min each) and incubated in 0.03% hydrogen peroxide (H2O2) for 20 min to quench endogenous peroxidase activity. After additional washes, the sections were incubated for 15 min in a blocking solution containing 2% nonfat dry milk in PBS. The sections were then incubated for 18 h at room temperature with rabbit anti-TH polyclonal antibody (1:1000; cat. no. E2L6M, Cell Signaling Technology, USA) or rabbit anti-α-Syn polyclonal antibody (1:1000; cat. no. E4U2F, Cell Signaling Technology, USA), diluted in 0.4% Triton X-100 prepared in PBS. After primary antibody incubation, the sections were washed and incubated for 2 h with a biotinylated anti-rabbit IgG secondary antibody (Vector Laboratories, USA) diluted in Triton X-100 solution. Sections were subsequently incubated for an additional 2 h with an avidin-biotin-peroxidase complex solution (Vectastain® Elite ABC Kit, Vector Laboratories, USA). Immunoreactivity was visualized using 3,3′-diaminobenzidine (DAB; Sigma-Aldrich, USA) in the presence of 0.01% H2O2 for 2 min in TH-stained sections and for 1 min in α-Syn-stained sections. After staining, the sections containing the regions of interest were mounted on gelatin-coated slides and dried at room temperature for 72 h. The slides were then rinsed in distilled water, dehydrated through graded ethanol solutions (70%, 95%, and 100%), cleared in xylene, and coverslipped. Images were acquired under bright-field illumination using a Nikon Eclipse Ni-E microscope (equipped with ×10 and ×25 objective lenses) and coupled to a Motic 5.0 digital camera.

Image analysis was performed using ImageJ (NIH) with the IHC Profiler plugin [48]. For each animal, four images from the same region of interest obtained from different histological sections were analyzed. Quantification was performed by an investigator who was blinded to the treatment allocation. For each brain region, images used for quantitative analysis were obtained from comparable anatomical levels across animals and experimental groups. No background subtraction, background color removal, or additional image processing was performed before analysis. All images were acquired and analyzed under identical conditions across experimental groups. The optical density score was calculated from staining intensity according to the IHC Profiler algorithm using the following formula: Score = (Low Positive × 1) + (Positive × 2) + (High Positive × 3). The mean score obtained from the four images was calculated for each animal and subsequently averaged within each experimental group. Data were normalized to the CTL-CTL group and used for statistical analysis [49, 50].

### Statistical Analysis

Data are expressed as the mean ± standard error of the mean (SEM). Before statistical analysis, normality was assessed using the Shapiro-Wilk test applied to the residuals of the statistical models. No significant deviations from normality were detected (*p *> 0.05), supporting the use of parametric statistical analyses. Outliers were identified using the ROUT method (*Q *= 1%) implemented in GraphPad Prism and excluded from the analysis. Statistical significance was set at *p *≤ 0.05.

OF and immunohistochemical data were analyzed using one-way ANOVA, followed by Tukey’s multiple-comparisons post hoc test. Catalepsy, oral movements, and tactile sensitivity data were analyzed using two-way repeated-measures ANOVA, with treatment as the between-subject factor and time as the within-subject factor, followed by Tukey’s post hoc test. When appropriate, the Greenhouse-Geisser correction was applied to the repeated-measures analyses. All statistical analyses and graphical representations were performed using GraphPad Prism version 8.0 (GraphPad Software, San Diego, CA, USA).

## Results

### CIN Attenuated Reserpine-Induced Sensorimotor Deficits in the Adhesive Removal Test

**Fig. 2:**
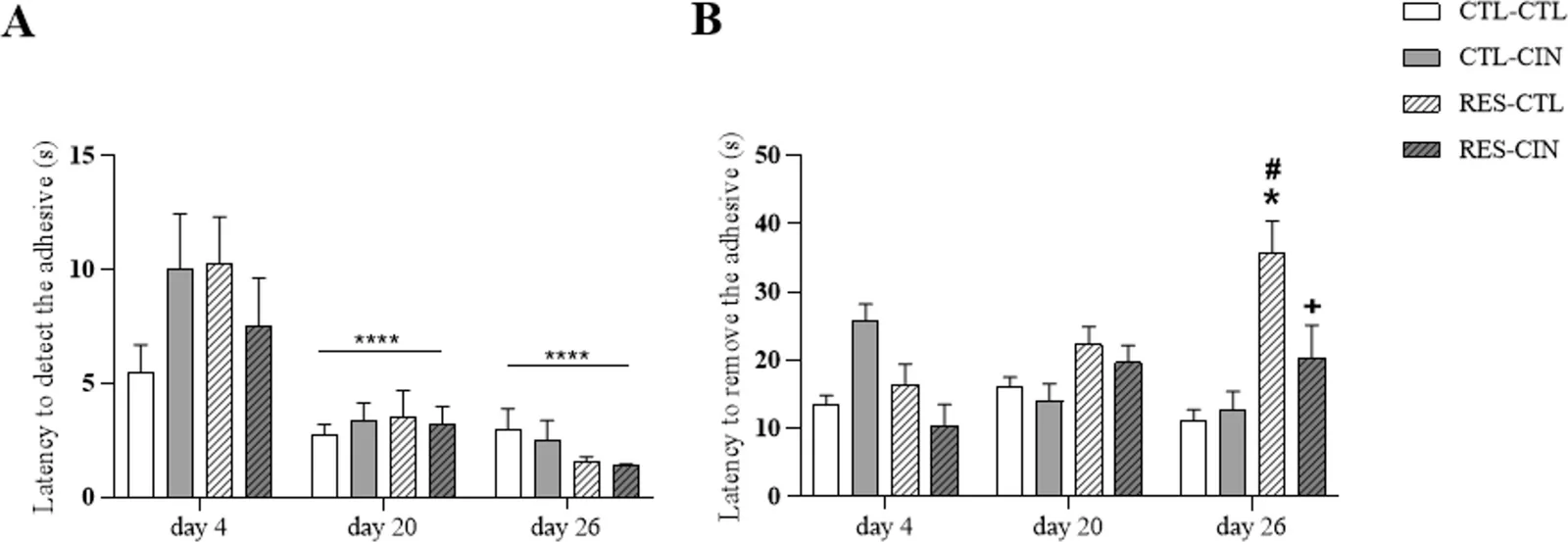
Effects of repeated CIN administration on sensorimotor performance in reserpine-treated mice, as assessed using the adhesive removal test. **A** Latency to detect the adhesive and **B** latency to remove the adhesive on treatment days 4, 20, and 26. *p ≤ 0.05 compared with the other groups at the same time point (*n *= 8/group). Data are expressed as the mean ± SEM (two-way repeated-measures ANOVA followed by Tukey’s post hoc test)

Two-way ANOVA repeated measures revealed a significant effect of time on adhesive detection latency [*F*(2,87) = 25.02, *p* < 0.05], whereas neither effect of the treatment [*F*(3,87) = 1.02, *p* = 0.44] nor the time × treatment interaction [*F*(6,87) = 1.11, *p* = 0.36] was significant. Tukey’s post hoc test showed that detection latency was higher on day 4 than on day 20 (*p* < 0.0001) and day 26 (*p *< 0.0001), but no significant difference between days 20 and 26 (*p* = 0.47) (Fig. 2A).

For adhesive removal latency, two-way ANOVA revealed significant effects of treatment [*F*(3,87) = 7.55, *p* < 0.05] and the time × treatment interaction [*F*(6,87) = 6.78, *p* < 0.05], whereas the main effect of time was not significant [*F*(2,87) = 1.39, *p* = 0.25]. Tukey’s post hoc test showed that, on day 26, the RES-CTL group exhibited longer adhesive removal latency than the other groups. In addition, the RES-CIN group showed shorter removal latency than the RES-CTL group (*p* < 0.05), indicating that CIN attenuated reserpine-induced sensorimotor impairment (Fig. 2B). Although the CTL-CIN group appeared to have a longer removal latency than the CTL-CTL group on day 4, this difference was not statistically significant (adjusted *p* = 0.09).

### CIN Modulated Locomotor and Exploratory Behavior in the Open Field Test

In the OF test, animals were evaluated on treatment days 10, 16, and 20. No significant group differences were observed in any parameter on day 10 (*p *> 0.05) (Table 1).

**Table 1:** Effects of repeated CIN administration on locomotor and exploratory behavior in the open field test in reserpine-treated mice

Day	Treatment	Total distance traveled (m)	Mean velocity (m/s)	Entries into the center zone	Distance traveled in the center zone (m)
10	CTL-CTL	15.77 ± 4.10	0.053 ± 0.01	38 ± 14	5.76 ± 2.76
	CTL-CIN	17.22 ± 4.35	0.060 ± 0.01	40 ± 11	5.49 ± 1.74
	RES-CTL	12.64 ± 5.67	0.042 ± 0.02	32 ± 18	4.24 ± 2.98
	RES-CIN	12.47 ± 5.25	0.042 ± 0.02	34 ± 10	3.96 ± 1.53
16	CTL-CTL	6.24 ± 3.51	0.021 ± 0.01	11 ± 10	1.38 ± 1.39
	CTL-CIN	8.31 ± 4.40	0.030 ± 0.01	18 ± 17	2.57 ± 2.65
	RES-CTL	2.38 ± 1.79*	0.008 ± 0.01*	3 ± 2*	0.32 ± 0.19*
	RES-CIN	2.59 ± 2.79*	0.009 ± 0.01*	4 ± 4*	0.38 ± 0.48*
20	CTL-CTL	7. 08 ± 4.84	0.024 ± 0.02	9 ± 8	1.17 ± 2.06
	CTL-CIN	9.07 ± 4.17	0.030 ± 0.01	24 ± 11	3.39 ± 2.44
	RES-CTL	4.45 ± 1.82*	0.015 ± 0.01*	8 ± 8*	0.92 ± 0.99*
	RES-CIN	4.00 ± 2.83*	0.013 ± 0.01*	9 ± 10*	0.92 ± 1.31*

Data are expressed as mean ± SEM. Each treatment day was analyzed separately using one-way ANOVA followed by Tukey’s multiple-comparisons test. *p ≤ 0.05 compared with the CTL-CTL group; all other pairwise comparisons were not significant (*p *> 0.05), *n *= 8/group

On day 16, significant group differences were observed in total distance traveled [*F*(3,32) = 7.57, *p* < 0.05], mean velocity [*F*(3,32) = 7.45, *p* < 0.05], number of entries into the center zone [*F*(3,32) = 4.56, *p* < 0.05], and distance traveled in the center zone [*F*(3,32) = 4.49, *p* < 0.05]. Tukey’s post hoc test showed that the RES-CTL group exhibited lower total distance traveled, mean velocity, number of center-zone entries, and distance traveled in the center zone than the CTL-CIN group (*p *< 0.05). The RES-CIN group also exhibited lower values for all four parameters than the CTL-CIN group (*p *< 0.05). No significant differences were observed between the RES-CTL and CTL-CTL groups or for the remaining pairwise comparisons (*p *> 0.05) (Table 1).

On day 20, significant group differences were also observed in total distance traveled [*F*(3,32) = 4.24, *p *< 0.05], mean velocity [*F*(3,32) = 4.08, *p *< 0.05], number of entries into the center zone [*F*(3,32) = 7.00, *p *< 0.05], and distance traveled in the center zone [*F*(3,32) = 4.30, *p *< 0.05]. Tukey’s post hoc test showed that the RES-CTL group exhibited lower total distance traveled, mean velocity, and distance traveled in the center zone than the CTL-CIN group (*p *< 0.05). The RES-CIN group also exhibited lower values for these three parameters than the CTL-CIN group (*p *< 0.05). For the number of entries into the center zone, significant differences were observed between RES-CTL and CTL-CIN and between RES-CIN and CTL-CIN (*p *< 0.05). No significant differences were observed between the RES-CTL and CTL-CTL groups or in the remaining pairwise comparisons (*p *> 0.05) (Table 1).

### CIN Attenuated Reserpine-Induced Catalepsy

**Fig. 3:**
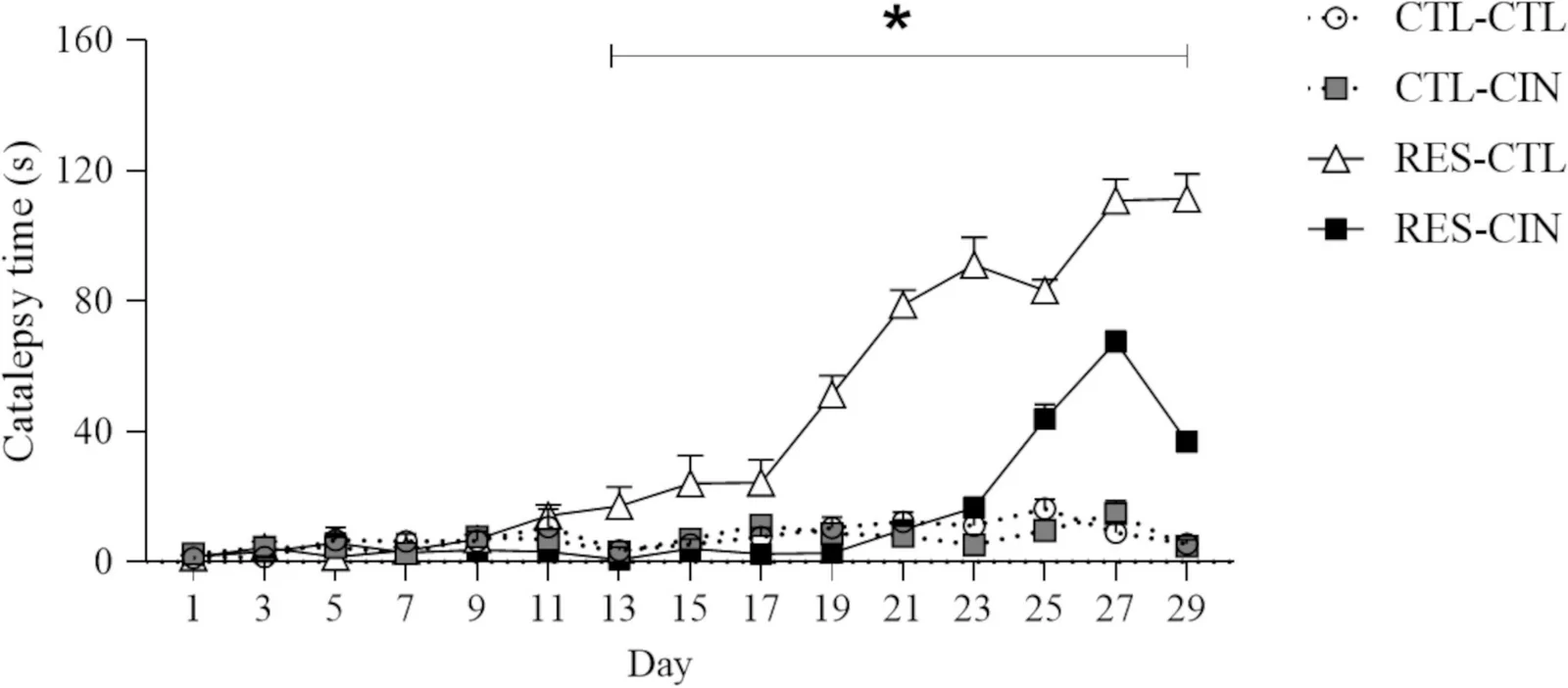
Effects of repeated CIN administration cataleptic behavior in reserpine-treated mice. Total duration of catalepsy across treatment days. *p ≤ 0.05 for the RES-CTL group compared with the other groups (*n *= 8). Data are expressed as the mean ± SEM (two-way repeated-measures ANOVA followed by Tukey’s post hoc test)

Two-way repeated-measures ANOVA revealed significant effects of time [*F*(14,43) = 95.60, *p* < 0.05], treatment [*F*(3,43) = 380.5, *p* < 0.05], and the time × treatment interaction [*F*(42,43) = 36.71, *p* < 0.05]. Tukey’s post hoc test showed that RES-CTL animals exhibited a progressive increase in catalepsy duration from day 13 onward compared with the control groups (*p* < 0.05), whereas the RES-CIN group showed reduced catalepsy duration from the same time point onward (*p *< 0.05), indicating that CIN attenuated reserpine-induced cataleptic behavior (Fig. 3).

### CIN Reduced Reserpine-Induced Vacuous Chewing Movements and Chin Tremors

**Fig. 4:**
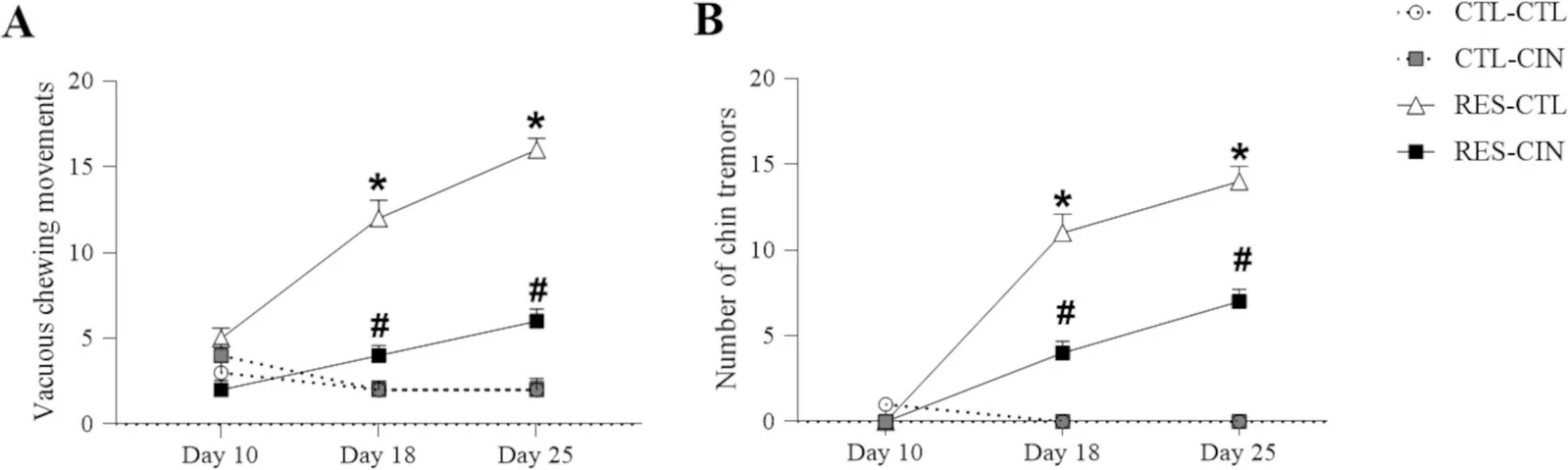
Effects of repeated CIN administration on oral movements in reserpine-treated mice. Frequencies of **A** vacuous chewing movements and **B** chin tremors on treatment days 10, 18, and 25. *p ≤ 0.05 compared with the other groups; #*p *≤ 0.05 compared with the RES-CTL group (*n *= 8). Data are expressed as the mean ± SEM (two-way repeated-measures ANOVA followed by Tukey’s post hoc test)

Two-way repeated-measures ANOVA of vacuous chewing movements revealed significant effects of time [*F*(2,87) = 21.39, *p* < 0.05], treatment [*F*(3,87) = 116.0, *p* < 0.05], and the time × treatment interaction [*F*(6,87) = 21.92, *p* < 0.05] (Fig. 4A). Tukey’s post hoc test showed that RES-CTL animals exhibited more vacuous chewing movements than the RES-CIN group from day 18 onward (*p* < 0.05), indicating that CIN reduced reserpine-induced vacuous chewing movements.

Similarly, analysis of chin tremors revealed significant effects of time [*F*(2,87) = 104.9, *p* < 0.05], treatment [*F*(3,87) = 178.7, *p* < 0.05], and the time × treatment interaction [*F*(6,87) = 53.54, *p* < 0.05]. Tukey’s post hoc test showed that RES-CTL animals exhibited more chin tremors than all other groups (*p* < 0.05). In addition, the RES-CIN group exhibited fewer chin tremors than the RES-CTL group (*p* < 0.05), indicating that CIN attenuated reserpine-induced chin tremors (Fig. 4B).

### CIN Attenuated the Reduction in Tyrosine Hydroxylase Immunoreactivity in the Substantia Nigra and Ventral Tegmental Area

One-way ANOVA revealed significant group differences in TH immunoreactivity in the substantia nigra pars compacta (SNpc) [*F*(3,16) = 20.08, *p *< 0.05] and ventral tegmental area (VTA) [*F*(3,16) = 7.62, *p *< 0.05], but not in the dorsal striatum [*F*(3,16) = 1.66, *p *= 0.21]. Tukey’s post hoc test showed that, in the SNpc, TH immunoreactivity was lower in the RES-CTL group than in the CTL-CTL and CTL-CIN groups (*p *≤ 0.05). TH immunoreactivity in the SNpc was also higher in the RES-CIN group than in the RES-CTL group (*p *≤ 0.05), suggesting that CIN attenuated the reserpine-induced reduction in TH immunoreactivity in this region.

**Fig. 5:**
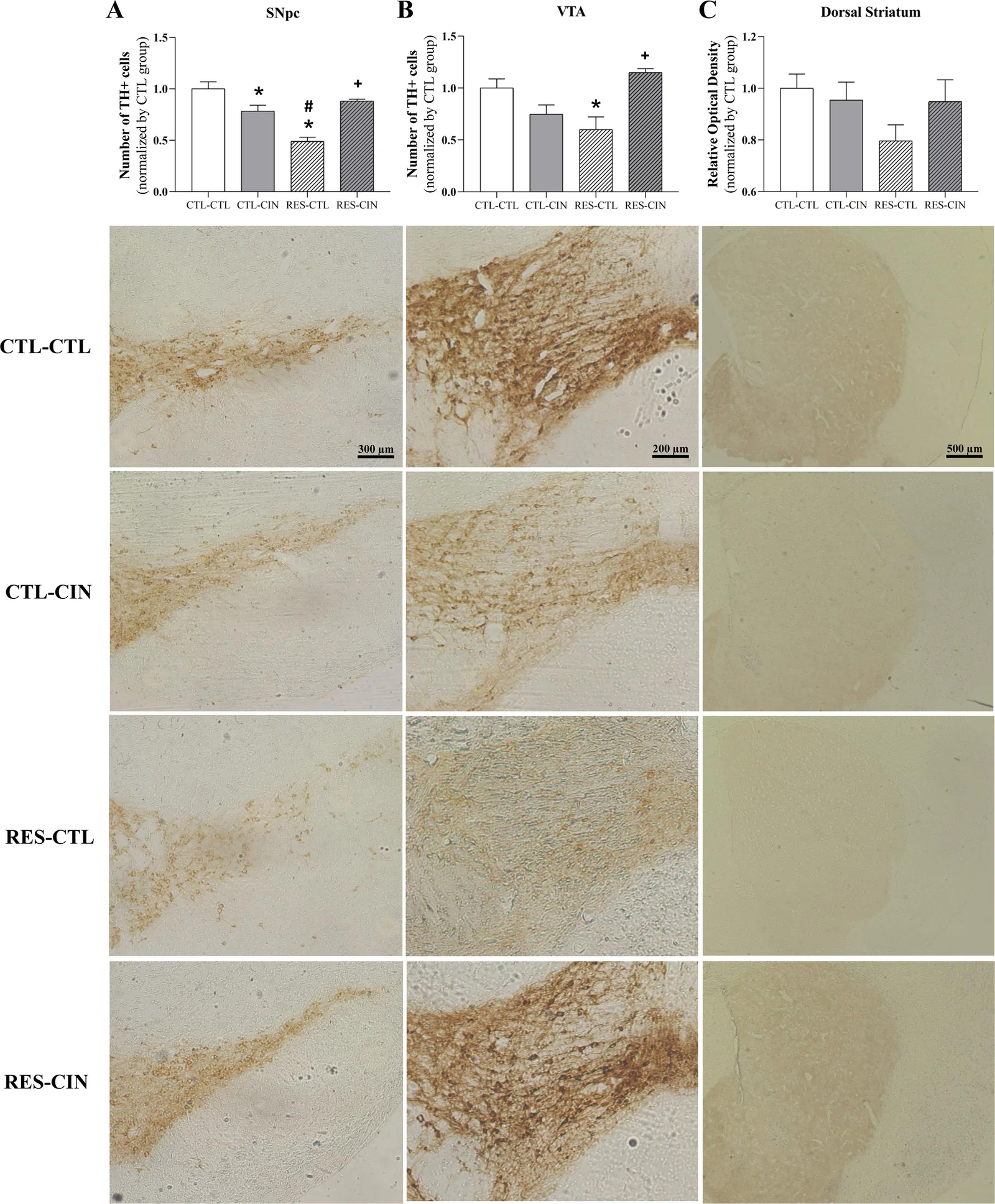
CIN attenuates the reserpine-induced reduction in TH immunoreactivity in midbrain dopaminergic nuclei. Quantification of TH immunoreactivity in the **A** substantia nigra pars compacta (SNpc), **B** ventral tegmental area (VTA), and **C** dorsal striatum. Representative TH-immunostained sections from each group are shown below the corresponding graphs. *p ≤ 0.05 compared with the CTL-CTL group; #*p *≤ 0.05 compared with the CTL-CIN group; + *p *≤ 0.05 compared with the RES-CTL group (*n *= 5). Data are expressed as mean ± SEM (one-way ANOVA followed by Tukey’s post hoc test)

In the VTA, TH immunoreactivity was lower in the RES-CTL group than in the CTL-CIN group and higher in the RES-CIN group than in the RES-CTL group (*p *≤ 0.05). No significant differences were observed in the dorsal striatum (Fig. 5).

### CIN Did Not Prevent the Reserpine-Induced Increase in Hippocampal α-Synuclein Immunoreactivity

**Fig. 6:**
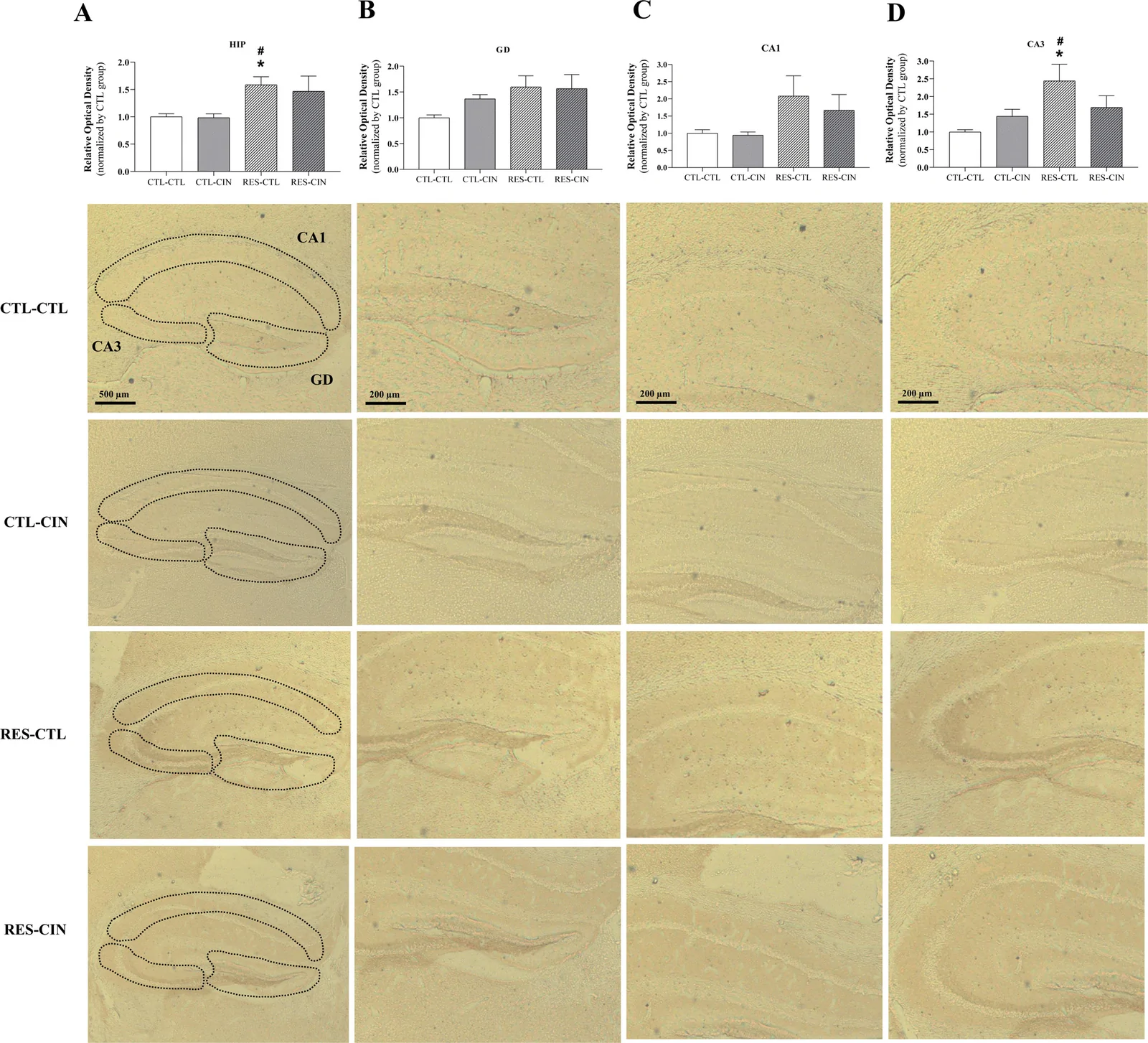
Effects of repeated CIN administration on hippocampal α-synuclein immunoreactivity in reserpine-treated mice. Relative optical density of α-synuclein in the **A** whole hippocampus (HIP), **B** dentate gyrus (DG), **C** CA1, and **D** CA3. Representative α-synuclein-immunostained sections from each group, with the hippocampal subregions outlined, are shown below. *p ≤ 0.05 compared with the CTL-CTL group; #*p *≤ 0.05 compared with the CTL-CIN group (*n *= 5). Data are expressed as the mean ± SEM (one-way ANOVA followed by Tukey’s post hoc test)

One-way ANOVA revealed significant group differences in α-synuclein optical density in the whole hippocampus [*F*(3,16) = 3.66, *p *< 0.05] and CA3 [*F*(3,16) = 3.90, *p *< 0.05], but not in the dentate gyrus [*F*(3,16) = 2.32, *p *= 0.11] or CA1 [*F*(3,16) = 2.10, *p *= 0.14]. Tukey’s post hoc test showed that the RES-CTL group exhibited higher α-synuclein immunoreactivity in the whole hippocampus and CA3 than the CTL-CTL and CTL-CIN groups (*p *≤ 0.05). No significant difference was detected between the RES-CTL and RES-CIN groups in any hippocampal region analyzed, indicating that CIN did not significantly modify hippocampal α-synuclein immunoreactivity under the present experimental conditions (Fig. 6).

## Discussion

The present study shows that β-cyclodextrin-complexed 1,8-cineole attenuated behavioral impairments induced by repeated RES administration and was associated with reduced alterations in dopaminergic markers in mesencephalic regions. CIN treatment reduced progressive catalepsy, vacuous chewing movements, and chin tremors while improving sensorimotor performance in the adhesive removal test. These behavioral effects were accompanied by attenuation of the reduction in TH immunoreactivity in the substantia nigra pars compacta and ventral tegmental area. These findings indicate that CIN attenuated functional impairments associated with monoamine depletion and support its further investigation as a neuroactive compound in experimental models of parkinsonism.

Among the experimental models used in PD research, the reserpine model has been widely employed because it reproduces several motor and neuropathological alterations associated with monoaminergic depletion. RES irreversibly inhibits vesicular monoamine transporter 2 (VMAT2) in the central nervous system, leading to progressive depletion of dopamine, serotonin, and noradrenaline [26, 51, 52]. This depletion induces hypolocomotion, rigidity, and abnormal oral movements [53], whereas prolonged administration has also been associated with reduced TH expression, neural dysfunction within nigrostriatal pathways, and increased α-Syn immunoreactivity [54, 55]. These features support the use of the RES model to investigate potential therapeutic interventions, including those based on natural compounds [28, 56].

1,8-Cineole (CIN), a monoterpene abundant in *Eucalyptus* species, and has been reported to exert anti-inflammatory [57–59], and antioxidant [31, 33] properties, with emerging evidence of neuroprotective-like effects under different experimental conditions. Furthermore, complexation with β-cyclodextrin enhances its stability, solubility and bioavailability while reducing volatility and improving its pharmacological profile [8, 39, 60]. These characteristics support the investigation of β-cyclodextrin-complexed CIN as a potential strategy for attenuating the behavioral and neurochemical alterations associated with parkinsonism. However, the present study did not directly assess oxidative stress, inflammatory mediators, or endogenous antioxidant pathways, and therefore these mechanisms cannot be established from the current data.

In the adhesive removal test, RES-treated animals showed sensorimotor deficits, as evidenced by increased adhesive removal latency, which may reflect impairments in sensorimotor integration and motor execution associated with monoaminergic dysfunction [17, 61, 62]. CIN treatment partially improved this parameter, suggesting attenuation of functional impairment despite chronic RES exposure. Detection latency was not affected by treatment but decreased across groups over time. This temporal reduction may reflect habituation or behavioral adaptation resulting from repeated exposure to the task rather than a treatment-specific improvement in tactile sensitivity. Accordingly, the detection-latency findings should not be interpreted as a treatment-specific effect. The motor component required for adhesive removal therefore appeared to be more sensitive to monoamine depletion than primary sensory detection [63, 64].

Motor-function assessment confirmed the progressive development of parkinsonian-like alterations following repeated RES administration. RES-treated animals exhibited increased catalepsy, vacuous chewing movements, and chin tremors, findings that are consistent with previous studies using monoamine-depletion models [47, 65, 66]. These behavioral alterations are commonly associated with dysfunction of basal ganglia dysfunction and impaired dopaminergic neurotransmission [29–31, 67]. CIN treatment significantly attenuated these abnormalities throughout the experimental period, suggesting a beneficial effect on RES-induced progressive motor impairment.

The reduction in cataleptic behavior observed in CIN-treated animals is particularly relevant because catalepsy is one of the most reproducible motor outcomes in RES-induced models of parkinsonism [51, 54]. Similarly, the attenuation of vacuous chewing movements and chin tremors suggests that CIN reduces the behavioral manifestations associated with dopaminergic dysfunction. Although the precise mechanisms underlying these effects were not investigated in the present study, previous evidence indicates that CIN can modulate biological processes involved in neural vulnerability, including oxidative stress and inflammatory responses [29–34, 57–59]. Antioxidant and anti-inflammatory actions therefore represent plausible explanations for the observed behavioral effects, but they should be regarded as literature-based hypotheses rather than mechanisms directly demonstrated in the present study.

Although CIN improved cataleptic behavior and abnormal oral movements, it did not significantly restore spontaneous locomotor activity in the OF test. This apparent discrepancy may reflect distinct neural substrates underlying these behavioral outcomes. Whereas catalepsy primarily reflects nigrostriatal dysfunction and motor rigidity, spontaneous locomotor activity is also influenced by motivational, emotional, and exploratory components, which may be more sensitive to the widespread monoaminergic depletion induced by RES [40, 51, 54].

An important finding of the present study was the attenuation of RES-associated reductions in TH immunoreactivity in the SNpc and the VTA. TH is the rate-limiting enzyme in dopamine synthesis and is widely used as a marker of the dopaminergic phenotype and functional status [68]. Alterations in TH immunoreactivity are commonly associated with changes in dopaminergic neurotransmission and have been reported in both clinical and experimental models of PD [68]. Consistent with previous studies using RES-induced models [69, 70], RES-treated animals exhibited reduced TH immunoreactivity in both the SNpc and VTA. In contrast, CIN-treated animals showed attenuated reductions in TH immunoreactivity within dopaminergic nuclei involved in motor and non-motor aspects of PD-related dysfunction.

The CIN-associated attenuation of reduced TH immunoreactivity was observed not only in the SNpc but also in the VTA. Although degeneration of the nigrostriatal pathway is classically associated with the motor manifestations of PD [71], increasing evidence indicates that the VTA is also affected during disease progression [18]. As the major source of mesocorticolimbic dopaminergic projections, the VTA contributes to motivational, cognitive, and affective processes that may be disrupted in PD [71]. The effects observed in both mesencephalic nuclei may therefore suggest that CIN influences dopaminergic beyond those directly involved in motor control.

Reduced TH immunoreactivity was also observed in the CTL-CIN group compared with the CTL-CTL group in the SNpc. The biological significance of this finding remains unclear because the present experimental design cannot determine whether this result reflects modulation of TH expression, alterations in the dopaminergic phenotype, or other biological processes. In contrast, under conditions of RES-induced monoamine depletion, CIN attenuated the reduction in TH immunoreactivity in both the SNpc and VTA. These findings suggest that the effects of CIN on TH immunoreactivity may depend on the underlying physiological context, an issue that warrants further investigation.

The attenuation of reduced TH immunoreactivity occurred in parallel with improvements in motor and sensorimotor performance. Although these findings do not establish a causal relationship, the association between TH immunoreactivity and behavioral improvement suggests that modulation of dopaminergic function may contribute to the effects observed after CIN treatment. No significant differences were detected in striatal TH immunoreactivity, suggesting that the effects of CIN may be more evident in dopaminergic cell bodies located within the mesencephalic nuclei than in terminal projections under the present experimental conditions. Similar discrepancies between somatic and terminal dopaminergic markers have been reported in experimental models of parkinsonism and may reflect distinct stages of pathological progression [68–70].

In addition to dopaminergic alterations, repeated RES administration increased α-Syn immunoreactivity in the hippocampus, particularly in the CA3 subregion. Although PD is primarily characterized by degeneration of the nigrostriatal dopaminergic pathways, α-Syn pathology is not restricted to these regions and has also been described in extranigral structures, including the hippocampus. Hippocampal α-Syn immunoreactivity was therefore included as a complementary histopathological marker to investigate whether repeated RES administration induced α-Syn-related alterations beyond the dopaminergic changes observed in the mesencephalon.

α-Syn is a presynaptic protein involved in synaptic vesicle dynamics and neurotransmitter release [7, 72], and its pathological accumulation is considered a central event in the pathophysiology of PD [7, 13]. According to Braak’s staging hypothesis, α-Syn pathology progressively extends from brainstem structures to limbic regions, including the hippocampus, where it is associated with cognitive dysfunction and neuronal vulnerability [5, 73, 74]. The increased hippocampal α-Syn immunoreactivity observed in the present study therefore indicates that repeated RES administration was associated with α-Syn-related alterations in this extranigral region. However, CIN treatment did not significantly alter hippocampal α-Syn immunoreactivity under the present experimental conditions. Thus, the behavioral improvements and attenuation of TH alterations observed in CIN-treated animals occurred without detectable changes in hippocampal α-Syn immunoreactivity. Although CIN has exerted neuroprotective effects in other experimental models [30, 75], including models involving protein-related pathological processes [2], evidence that it modulates α-Syn accumulation remains limited.

Under the present experimental conditions, CIN attenuated the reserpine-induced changes in TH immunoreactivity but did not significantly modify hippocampal α-Syn immunoreactivity. These differential findings should be interpreted cautiously because the present data do not establish whether CIN directly modulates α-Syn-related processes or identify the molecular pathways underlying its effects. Further studies specifically assessing α-Syn expression, aggregation, and regional distribution are required to determine whether these processes are influenced by CIN.

Several limitations of the present study should be acknowledged. Although the behavioral and immunohistochemical outcomes support the beneficial effects of CIN, the biochemical pathways potentially involved in these effects were not directly investigated. Markers of oxidative stress, neuroinflammation, mitochondrial dysfunction, and dopamine metabolism were not evaluated; therefore, mechanistic interpretation remains limited. Furthermore, only a single CIN dose was evaluated. Although this dose was selected based on previous evidence of pharmacological activity, the use of a single-dose design precluded assessment of dose-response relationships and prevents the determination of the optimal therapeutic range. Consequently, it remains unclear whether lower doses would produce similar effects or whether higher doses would provide additional benefits or cause adverse effects. Future studies should therefore investigate multiple CIN doses to establish its therapeutic window and efficacy and safety profiles in experimental models of parkinsonism. Future studies should also directly examine whether the behavioral and histological effects observed after CIN treatment are associated with modulation of oxidative stress, inflammatory signaling, mitochondrial function, dopamine metabolism, and endogenous antioxidant defenses.

An additional limitation of the present study is that TH immunoreactivity was used as an indirect marker of dopaminergic phenotype and function. Because TH staining alone does not permit direct assessment of neuronal survival, the present findings cannot determine whether attenuated reductions in TH immunoreactivity reflect preservation of TH expression, prevention of dopaminergic neuronal loss, or both. Future studies using stereological approaches, NeuN co-labeling, or complementary markers of neurodegeneration are needed to determine whether CIN exerts direct neuroprotective effects on dopaminergic neurons. In addition, α-Syn immunoreactivity was evaluated only in the hippocampus. The present findings therefore do not support conclusions regarding α-Syn pathology within the nigrostriatal pathway, particularly in the SNpc and striatum. Future studies specifically designed to evaluate these regions will be important to further characterize the effects of CIN on α-Syn pathology.

## Conclusion

In summary, β-cyclodextrin-complexed 1,8-cineole attenuated motor and orofacial impairments and improved sensorimotor performance in a RES-induced mouse model of parkinsonism. These behavioral effects were accompanied by attenuation of the reduction in TH immunoreactivity in mesencephalic dopaminergic regions. Previous studies indicate that CIN has antioxidant and anti-inflammatory properties; however, these mechanisms were not directly investigated and should therefore be regarded as plausible hypotheses rather than demonstrated explanations for the present findings. Despite the absence of a detectable effect on hippocampal α-Syn immunoreactivity, the behavioral and immunohistochemical outcomes support further investigation of CIN as a neuroactive compound in experimental models of parkinsonism.

## Data Availability

All data supporting the findings of this study are available within the paper.
